# Daily Heart Rate Variability Indices in Subjects with and Without Metabolic Syndrome Before and After the Elimination of the Influence of Day-time Physical Activity

**DOI:** 10.3390/medicina55100700

**Published:** 2019-10-17

**Authors:** Anžela Slušnienė, Aleksandras Laucevičius, Petras Navickas, Ligita Ryliškytė, Vytautas Stankus, Albinas Stankus, Rokas Navickas, Ieva Laucevičienė, Vytautas Kasiulevičius

**Affiliations:** 1 State Research Institute Centre for Innovative Medicine, LT-08406 Vilnius, Lithuania; slusniene.anzela@gmail.com (A.S.); aleksandras.laucevicius@mf.vu.lt (A.L.); albinas.stankus@gmail.com (A.S.); rokas.navickas@gmail.com (R.N.); 2 InMedica, LT-08406 Vilnius, Lithuania; 3 Faculty of Medicine, Vilnius University, LT-03101 Vilnius, Lithuania; ligita.ryliskyte@santa.lt (L.R.); ieva.lauceviciene@santa.lt (I.L.); vytautas.kasiulevicius@santa.lt (V.K.); 4 Centre of Cardiology and Angiology, Vilnius University Hospital Santaros Klinikos, LT-08661 Vilnius, Lithuania; 5 Kaunas University of Technology, LT-44249 Kaunas, Lithuania; vytautas.stankus@ktu.lt; 6 Centre of Rehabilitation, Vilnius University Hospital Santaros Klinikos, Physical and Sports Medicine, LT-08661 Vilnius, Lithuania

**Keywords:** metabolic syndrome, heart rate variability, physical activity, hypertension

## Abstract

*Background and Objectives:* The available research shows conflicting data on the heart rate variability (HRV) in metabolic syndrome (MetS) subjects. The discrepancy suggests a methodical shortcoming: due to the influence of physical activity, the standard measuring of HRV at rest is not comparable with HRV assessment based on 24 h Holter monitoring, which is preferred because of its comprehensiveness. To obtain a more reliable measure and to clarify to what extent HRV is altered in MetS, we assessed a 24 h HRV before and after the elimination of the influence of physical activity. *Materials and Methods:* We investigated 69 metabolic syndrome (MetS) and 37 control subjects, aged 50–55. In all subjects, 24 h monitoring of electrocardiogram, blood pressure, and actigraphy profiles were conducted. To eliminate the influence of day-time physical activity on RR intervals (RRI), a linear polynomial autoregressive model with exogenous terms (ARX) was used. Standard spectral RRI analysis was performed. *Results:* Subjects with MetS had blunted HRV; the diurnal SDNN index was reliably lower in the MetS group than in control subjects. The elimination of the influence of physical activity did not reveal a significant HRV change in long-term indices (SDNN, SDANN, and SD2), whilst adjacent RRI values (RMSSD, pNN50, and SD1) and SDNN index significantly increased (*p* < 0.001). An increase in the latter indices highlighted the HRV difference between the MetS and control groups; a significant (*p* < 0.001) decrease of all short-term HRV variables was found in the MetS group (*p* < 0.01), and low-frequency spectral components were less pronounced in the MetS group. *Conclusion:* The application of a polynomial autoregressive model in 24 h HRV assessment allowed for the exclusion of the influence of physical activity and revealed that MetS is associated with blunted HRV, which reflects mitigated parasympathetic tone.

## 1. Introduction

The metabolic syndrome (MetS) is an exceedingly prevalent condition that affects 20–40% of the middle-aged population globally [1,2]. This common condition is closely linked to an increased incidence of diabetes and has a detrimental effect on cardiovascular outcomes [3]. Most subjects with confirmed MetS have elevated blood pressure [4,5] and sympathetic overactivity [6,7]. This MetS component is associated with disturbed circadian blood pressure and decreased heart rate variability (HRV), which are linked to elevated sympathetic nervous system activity [8,9]. The latter, at least in part, is responsible for the accelerated development of unfavorable cardiovascular outcomes, type 2 diabetes mellitus [10], and sudden cardiac death [11]. Although MetS is present in 20–40% of the middle-aged population globally [10,11], altered autonomic nervous system (ANS) balance is still not included as the MetS defining component. Furthermore, it is not yet established how it is influenced by a physically active or sedentary lifestyle [12,13].

HRV studies that investigated autonomous heart rate (HR) modulation in various dynamic and/or stationary states showed ambiguous results. Short-term HR recordings obtained under laboratory conditions [14] have shown that hypertension (AH) is shown to be associated with lower HR sub-frequencies than high-frequencies that are associated with SNS activation [6,15]. These observations suggest that AH is associated with increased cardiac sympathetic activity and decreased cardiac parasympathetic activity. However, it ought to be emphasized that a change in a subject’s physical activity may lead to a variation of the measured variables [16], which makes it difficult to assess the dependence of autonomic nervous system equilibrium on existing pathology in outpatient settings. 

The aforementioned association is further altered when AH is accompanied by MetS, in which case short HR recordings reveal the imbalance of HRV indicators also in daily measurements—changes in ultra-low frequency (ULF) and very low frequency (VLF) spectrum bands [17,18]. In addition, outpatient day-to-day HR monitoring, due to environment-associated changes and uninhibited behavior, will cause many different HR reactions [12,19]. These diverse conditions prevent the results of the study to be precisely defined. We suggest that the assessment of additional physiological indicators that are linked to HR modulation is needed to explain the interdependence of ongoing processes and the role of ANS. Physical activity (PhA) has the greatest impact on HRV and can be recorded by an accelerometer [12]. However, the accelerometers’ signals only provide information about ongoing acceleration and its relative amplitude, hence making it very complicated to associate it with ANS modulated HRV. In this context, we aimed to investigate the daily heart rate variability (HRV) before and after elimination of the influence of physical activity fluctuations on HRV in individuals with and without metabolic syndrome (MetS) in groups of the same age, comparing day-time and night-time data.

## 2. Materials and Methods

### 2.1. Population

Altogether, we examined 106 middle-aged (50–55 years old) subjects. The investigation group consisted of 69 subjects with confirmed metabolic syndrome (according to the modified National Cholesterol Education Program, Adult Treatment Panel III (NCEP-ATP III) criteria) [20], in all of whom blood pressure (BP) of more than 130/85 mmHg was present, or they were treated with antihypertensive drugs. The control group consisted of 37 subjects of the same age without MetS and whose BP was less than 130/85 mmHg. All subjects were recruited from the ongoing Lithuanian High Cardiovascular Risk (LitHiR) primary prevention program [4,5].

### 2.2. Study Protocol and Measurements Used

**Figure 1:**
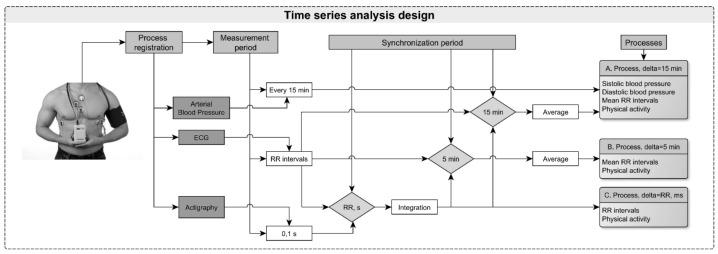
The sequence of investigation. ECG: electrocardiogram; RR: R-R intervals.

In all subjects, ambulatory 24 h monitoring was initiated between 7 am and 10 am. Daily electrocardiogram (ECG), blood pressure (BP) and actigraphy values were monitored with certified devices (ABPM-05 with “Card(X)plore”, Meditech Ltd., Hungary) placed on the patient’s left arm, whilst the actigraph was placed on the chest. The actigraph recorded whole-body movements that occurred during PhA. Three ECG signals were digitized with a discretization frequency 300 Hz/channel and two actigraphy channels with 10 Hz/channel. The day-time frequency of BP measurements was set at an interval of 15 min (07:00–23:00), and the night-time with an interval of 15–30 min (23:00–07:00). RR intervals (RRI) were determined from the simultaneous recordings of the 24 h Holter ECG (Figure 1). 

The study protocol was approved by the Vilnius Regional Biomedical Research Ethics Committee and written informed consent was obtained from all participants.

### 2.3. Data Analysis

A specific software (LabVIEW, National Instrument, USA) was used to remove all motion artifacts, premature beats, and all other artifacts of unknown origin from the ECG. The analysis was carried out separately for day-time (from 9 am to 9 pm) and night-time records (sleeping period was determined according to a subject’s diary). 

### 2.4. Sequence Transformations

**Figure 2:**
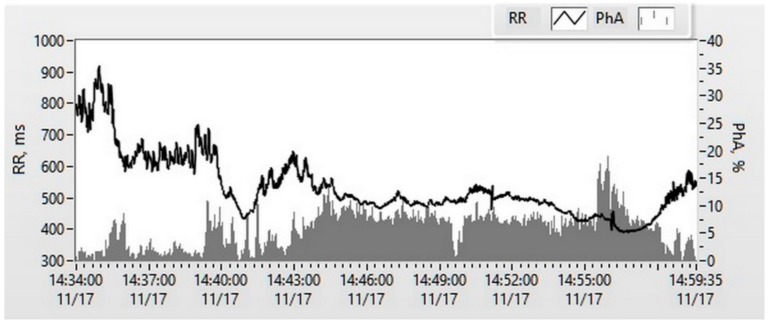
An example of a study patient’s actigraphy recording; time series of a 30 min segment of RRI and physical activity. RR: R-R intervals, ms; PhA: physical activity, %.

Dedicated software (LabVIEW, National Instrument, USA) was used for the analysis of the relationship between the processes with the different discretized frequencies. Constant components from both actigraphy processes were eliminated with filters. The magnitude of every (10 Hz) physical activity vector (VAG) was calculated from **x** and **y** coordinate values of the actigraph’s signals using the Euclidean norm (square root of both **x** and **y** square sums). All VAG were synchronically summed in each of RRI, and the sum was divided by the RRI duration (Figure 2). The resulting VAG values were normalized to the highest daily value and expressed as a percentage. For the time series analysis of the obtained RRI and VAG, the series was resampled by the cubic Hermite interpolation method [21] with the period equal to 0.5 s.

### 2.5. Time-Domain and Frequency-Domain Analysis of RRI 

**Figure 3:**
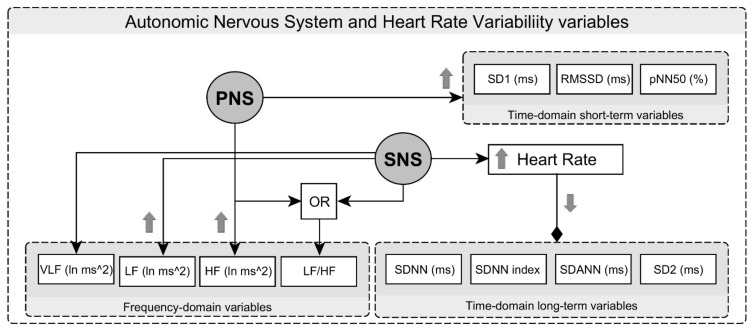
Heart rate variability indices characterizing the autonomic nervous system. Arrows indicate a decrease (↓) or increase (↑) in indicator activity. SNS: Sympathetic nervous system; SDNN: standard deviation of all normal to normal RRI; SDANN: standard deviation of 5-min RRI average; SDNN index: mean of the standard deviation of all 5-min RRI segments in 24 h; SD2: long-term beat-to-beat RRI variability from the Poincaré plot (length); PNS: parasympathetic nervous system; rMSSD: the square root of the mean of the sum of the squares of differences between adjacent RRI; SD1: short-term beat-to-beat RRI variability from the Poincaré plot (width); pNN50: the percentage of intervals >50 ms different from preceding interval; LF: low frequency; HF: high frequency; LF/HF: the ratio between LF and HF power.

HRV analysis was performed in accordance with the Task Force of the European Society of Pacing and Electrophysiology [22] using the Fast Fourier transform method. Long-term variability characterizing sympathetic nervous system (SNS) activity was assessed by the following variables: standard deviation of all normal to normal RRI (SDNN), standard deviation of 5-min RRI average (SDANN), mean of the standard deviation of all 5-min RRI segments in 24 h (SDNN index), and long-term beat-to-beat RRI variability from the Poincaré plot (length) (SD2). Short-term variability that characterizes the parasympathetic nervous system (PNS) was assessed by the square root of the mean of the sum of the squares of differences between adjacent RRI (rMSSD), short-term beat-to-beat RRI variability from the Poincaré plot (width) (SD1), and the percentage of intervals >50 ms different from preceding interval (pNN50) (Figure 3). 

The spectral components of HRV were analyzed by means of VLF (0.003–0.04 Hz), low frequency (0.05–0.15 Hz) (LF), and high frequency band (0.15–0.5 Hz) (HF) units. The ratio between LF and HF power (LF/HF) was calculated as well.

### 2.6. Assessment of Physical Activity-Related HRV Changes

In order to eliminate the influence of PhA on HRV measurements, we used a linear polynomial autoregressive model with exogenous terms (ARX) model. The identification method for the ARX model is the least-squares method, which solves linear regression equations in the analytic form [23]. The following equation shows the form of the ARX model.

A(z)y(k) = B(z)u(k) + e(k),

where A(z) and B(z) are polynomials with respect to the backward shift operator z^−1^, u(k) is the measuring inputs of the VAG series, e(k) is the system residual part, y(k) is the measuring outputs of RRI. The following are the time domain equations for the ARX model.

y(k) − a1y(k-1) + ⋯ + any(k-n) = b0u(k) + ⋯ + b1u(k-1) bm(k-m) + e(k),

where n is the A order; m is the B order and e(k) is the system disturbance.

**Figure 4:**
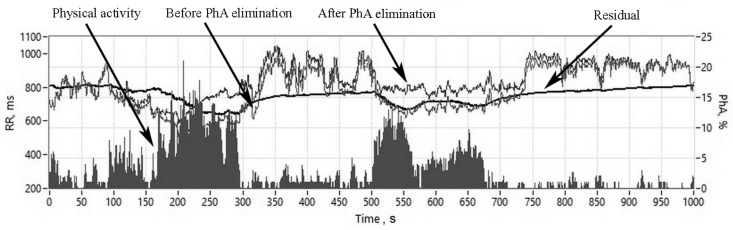
An exemplary sequence of PhA, RRI before and after the PhA influence elimination procedure. RR: R-R intervals, ms; PhA: physical activity, %.

We used a transfer function derived from the ARX model, where, based on the VAG signal, a new RRI response signal was obtained (Figure 4). In order to validate the model, a cross-correlation analysis between residual signals, e(k), and VAG signals, u(k), was performed. The model would have been deemed invalid if the residual and VAG signals correlated; if the cross-correlation was not zero, it would have suggested that the model could not capture all determinate data variations.

For the residual RRI sequence, a repeated HRV analysis was performed. Data analysis was carried out separately for day and night periods. The onset of sleep and its duration were assessed for each subject individually.

### 2.7. Statistical Analysis

Data analysis was executed using the IBM SPSS Software 21.0 version (SPSS, Chicago, IL, USA) and the data are presented as mean ± standard deviation. All *p*-values were two-sided. The comparison between quantitative measurements was performed using the Student’s *t*-test with p-values of 0.05, with less being considered statistically significant. The dependent sample t-test was applied to determine the difference between variables before and after the elimination of PhA influence. The χ^2^ test was used to evaluate the differences between categorical variables. Pearson correlation coefficient was used to measure the relationships among continuous measures. We used the GLM Multivariate procedure, which is based on the analysis of the regression and dispersion of dependent variables for one or more factor variables or covariates to evaluate or eliminate the effects of individual variables.

Ethical approval was obtained from the Vilnius Regional Biomedical Research Ethics Committee and written informed consent was obtained from all participants (No. 158200-17-889-400).

## 3. Results

### 3.1. Baseline Clinical Characteristics

There were no differences in age and gender between the investigation and control groups (Table 1). In subjects with MetS, components were distributed as follows: Increased arterial blood pressure and increased waist circumference was present in all 69 subjects (100%); low high-density lipoprotein cholesterol levels were found in 55 (80%), elevated fasting triglyceride levels in 44 (63.8%), and impaired fasting glucose in 38 (55.1%) subjects; diabetes was present in 10 (14.5%) of MetS subjects. A significant difference between MetS and control groups was observed with respect to all risk factors. 

Clinical assessment showed that blood pressure stage I and II were uniformly distributed in subjects with MetS (46.6% and 53.6% respectively). The mean 24 h, diurnal/nocturnal systolic and diastolic blood pressure was significantly higher in the MetS group (*p* < 0.001). More than two thirds (47, 68.1%) of MetS group subjects were on antihypertensive medication—17 (24.6%) were on beta-blockers, 35 (50.7%) on ACE inhibitors, 10 (14.5%) on angiotensin receptor blockers, 17 (24.6%) on calcium channel blockers, 16 (23.2%) on diuretics, and 38 (55.1%) used other drugs.

### 3.2. Heart Rate Variability Characteristics Before the Elimination of Physical Activity Influence

Heart rate variability data are presented in Table 2. There was no significant difference in SDNN between the study group and controls. However, the analysis of the 5-min RRI segments revealed that the SDNN index is reliably lower in the MetS group (47.8 ± 13.0 ms vs 53.8 ± 14.1 ms, *p* < 0.05), whereas SDANN did not differ significantly. The analysis of even shorter RRI segments calculating the variability of adjacent RRI values (RMSSD and pNN50) showed that these indices were lower in the MetS group. This trend is observed in the MetS group after applying the Poincaré distribution. The short-term beat-to-beat RRI variability from the Poincaré plot (width) SD1 was 14.2 ± 5.4 ms in the control group and 12.9 ± 4.9 ms in the MetS group (*p* > 0.05). The long-term beat-to-beat RRI variability from the Poincaré plot (length) SD2 was respectively 151.6 ± 37.5 ms vs 147.9 ± 37.0 ms (*p* > 0.05). These time-domain data indicate that subjects with MetS exhibit a tendency to decrease in HRV.

The frequency-domain analysis showed that the RRI variability in the VLF bandwidth did not differ between the groups. Significant difference was evident in the LF bandwidth: 5.86 ± 0.61 ln ms^2^ in control group vs 5.51 ± 0.59 ln ms^2^ in MetS (*p* < 0.01). In the HF bandwidth, only a downward trend was observed in the MetS group. Consequently, the LF/HF ratio was reliably lower in the MetS group (3.62 ± 1.35 vs 3.04 ± 1.37, *p* < 0.05), indicating that subjects with MetS exhibit a decrease in day-time variability of HR. 

During the night-time period, when subjects were in the nonmotile state, slow HR was present in both groups, and all RRI time-domain variables reliably differed from the day-time measures. The night-time SDNN decreased to 93.0 ± 31.1 ms (*p* < 0.05) in the control group, and to 90.0 ± 26.4 ms (*p* < 0.05) in the MetS group, whilst the SDNN index in the control group did not change, and in the MetS group, increased to 54.3 ± 16.7 ms (*p* < 0.05). Furthermore, in the control and study groups, SDANN sharply decreased to63.2 ± 20.0 ms and 64.4 ± 20.9 ms (*p* < 0.001), respectively, indicating that the effect of the sympathetic nervous system on heart rate fluctuation decreased during the night. 

The difference between adjacent RRI was very high (*p* < 0.001): during the night-time, RMSSD increased to 27.9 ± 10.7 ms and 27.8 ± 11.8 ms, and pNN50 to 8.9 ± 8.6% and 9.4 ± 9.5%. Furthermore, Poincaré plot indicators changed similarly. This dynamic allows us to state that parasympathetic HR regulation was equally highly expressed in both groups overnight.

The frequency-domain analysis of the night-time RRI segments showed different results. Firstly, the variability of RRI at night, as compared to the day-time measures, increased only in the high-frequency band: in the control group, from 4.66 ± 0.77 ln ms^2^ to 5.24 ± 0.87 ln ms^2^ (*p* < 0.01), whereas in the MetS group, from 4.50 ± 0.81 ln ms^2^ to 5.13 ± 0.92 ln ms^2^ (*p* < 0.01). Secondly, among subjects with MetS the VLF variable decreased to 7.52 ± 0.58 ln ms^2^ (*p* < 0.05). Thirdly, in contrast to the control group, the magnitude of LF in the MetS group has decreased statistically significantly. Likewise, the decrease of nocturnal LF/HF ratio was more pronounced in the MetS group (1.95 ± 1.16 (*p* < 0.001) vs 2.15 ± 1.03 (*p* < 0.001)). To summarize, the relationship between SNS and PNS effects on night-time HR varied, and day-time LF/HF differences between the groups disappeared during the night.

**Figure 5:**
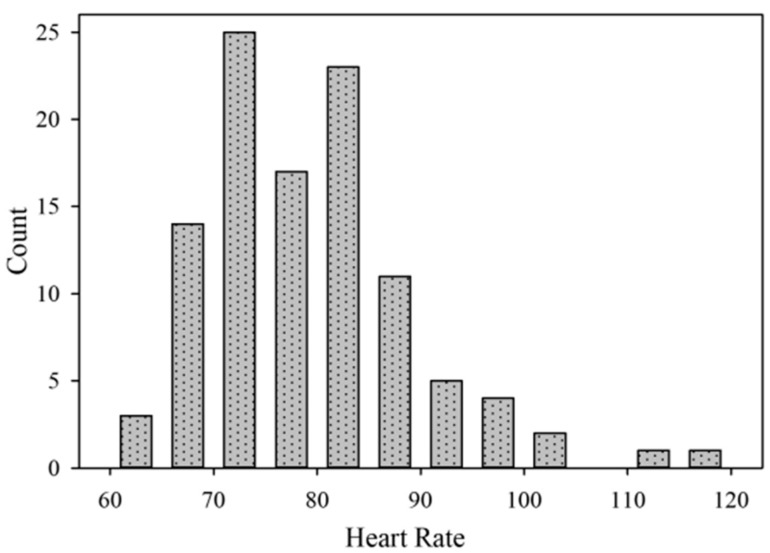
Pulse rate histogram of all subjects (*p* = 0.51 between groups).

We have noticed that there were many individuals whose daily pulse rate was higher, which is known to reduce HRV. The diurnal HR distribution analysis showed that the average pulse rate of all subjects during the day was higher than 60 bpm and reached up to 120 bpm (Figure 5).

**Figure 6:**
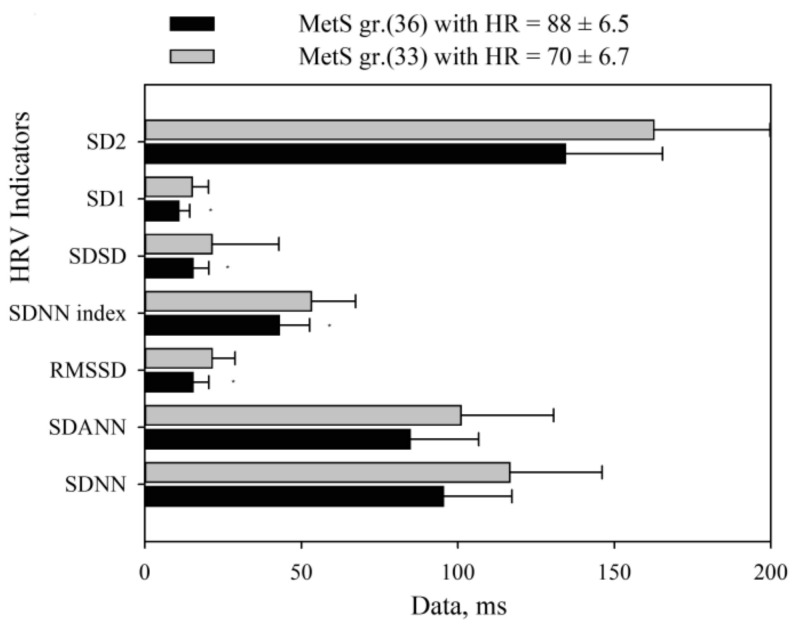
HRV time components association with heart rate. HRV: heart rate variability; MetS group: subjects with MetS (confirmed according to the modified NCEP-ATP III criteria) and AH; HR: heart rate; SDNN: standard deviation of all normal to normal RR intervals; SDANN: standard deviation of 5-min average RRI; RMSSD: the square root of the mean of the sum of the squares of differences between adjacent RRI; SDNN index: mean of the standard deviation of all 5-min RRI segments in 24 h; SDSD: standard deviation of differences between adjacent RRI intervals; SD1: short-term beat-to-beat RRI variability from the Poincaré plot (width); SD2: long-term beat-to-beat RRI variability from the Poincaré plot (length).

The latter result necessitated an additional analysis, which showed that HRV and pulse rate were highly interdependent: more frequent HR was associated with lower HRV. The correlation coefficient between the averaged RRI and HRV time-domain components varied from r = 0.39 to r = 0.53. This relationship indicates that the higher the SNS tone level (lower RRI), the lower the HRV (Figure 6). In addition, all HRV indices were reliably lower among subjects with increased HR than in the group with a less frequent HR (*p* < 0.001). 

A subsequent GLM Multivariate analysis confirmed that the HRV daily indices (RMSSD, SDSD, and SD1) and SDNN index were reliably (*p* < 0.003) lower in the MetS group than in control subjects.

The heart rate histogram demonstrates that the shift of all heart rate averages from 60 bpm to tachycardia is associated with the dominance of the day-time sympathetic nervous system and a subsequent decrease of HRV variables: SD1, SDSD, RMSSD, and SDNN index (Figure 6).

### 3.3. Heart Rate Variability Characteristics after Elimination of Physical Activity Influence

During the 24 h Holter monitoring in the outpatient setting, subjects are constantly exposed to physical activity. Hence, this raises the question of whether it is possible to eliminate the influence of this physical activity which is recorded by an actigraph, and to bring the HRV assessment closer to the measurements obtained under stationary conditions in a laboratory. Therefore, in the next step, we analyzed the daily HRV after the elimination of the influence of physical activity. Our analysis included a comparison of the individuals with and without metabolic syndrome, and of the day- and night-time HRV data.

**Figure 7:**
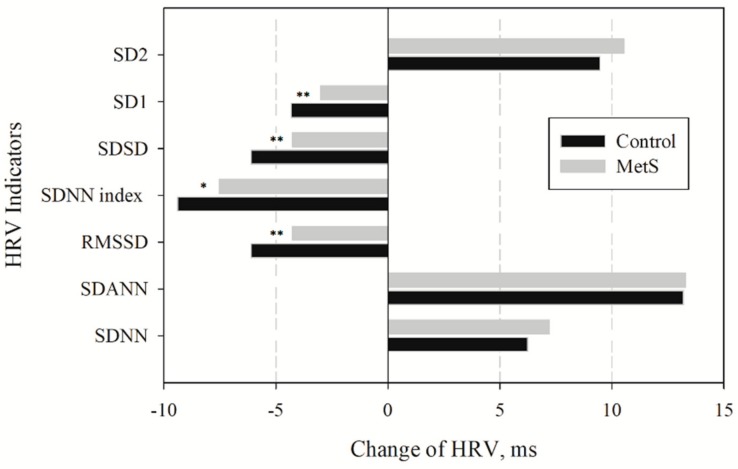
Time-domain HRV component’s dynamic before and after PhA elimination. * *p* < 0.05; ** *p* < 0.01, between control and MetS group. HRV: heart rate variability; MetS gr.: subjects with MetS (confirmed according to the modified NCEP-ATP III criteria) and AH; Control: subjects without MetS and AH; HR: heart rate; SDNN: standard deviation of all normal to normal RR intervals; SDANN: standard deviation of 5-min average RRI; RMSSD: the square root of the mean of the sum of the squares of differences between adjacent RRI; SDNN index: mean of the standard deviation of all 5-min RRI segments in 24 h; SDSD: standard deviation of differences between adjacent RRI intervals; SD1: short-term beat-to-beat RRI variability from the Poincaré plot (width); SD2: long-term beat-to-beat RRI variability from the Poincaré plot (length).

Our results have shown that an actigraph can be used to ascertain the change in HR associated with physical activity. First of all, this association is confirmed by the relatively high negative correlation between RRI and physical activity (r = −0.44 ± 0.13), which has decreased after eliminating the influence of PhA (r= −0.12 ± 0.2). Since PhA occurs during the day-time period, only day-time HRV indicators were analyzed. HRV data after PhA influence elimination is presented in Table 3 and Figure 7. A statistically significant change (presented as delta mean) was observed with respect to each variable (*p* < 0.001).

We observed that, after PhA influence elimination, the mean RRI changed by −62.3 ms (*p* < 0.001) in the control group, and by −75.6 ms in the MetS group (*p* < 0.001, see Table 2 and Table 3). Hence the elimination of PhA influence reduced the overall effect of SNS on heart rate. In both groups, long-term indices (SDNN, SDANN, and SD2) did not change. By contrast, RRI segments of adjacent RRI values (RMSSD, pNN50, and SD1) and 5-min RRI segments (SDNN) have significantly increased in both groups (*p* < 0.001). An increase in the latter indices highlighted the HRV difference between the MetS and control groups: a significant (*p* < 0.001) decrease of all short-term HRV variables was found in the MetS group (*p* < 0.01).

After the elimination of PhA influence, the frequency-domain analysis did not reveal any significant change in the VLF bandwidth, whereas in the LF bandwidth variability decreased, even if the difference between the groups remained significant (*p* < 0.01). HF bandwidth variability was not applicable for analysis since respiratory movements are inevitably recorded by accelerometers and fall within the same frequency bandwidth, thus producing PhA unrelated artifacts.

In sum, the elimination of physical activity-related SNS influence on HRV uncovers more pronounced short-term parasympathetic fluctuations in controls, but not MetS subjects. Our results, firstly indicate the real magnitude of the blunted HRV in the middle-aged MetS subjects with hypertension. Secondly, we demonstrated that the blunted HRV in these subjects is accounted for by the mitigation of parasympathetic tone.

## 4. Discussion 

The cluster of cardiovascular and metabolic risk factors—abdominal obesity, specific dyslipidemia, impaired glucose metabolism, and hypertension—are referred to as “metabolic syndrome”, which is related to increased risk of diabetes and cardiovascular diseases [3,24]. In their review, Stuckey et al. [18] emphasized that obese subjects have autonomous dysfunction during rest, yet little is still known about the effect of obesity on autonomic cardiac responses to mental and physical activity [25]. Consistent with the published data [26], our results showed that middle-aged subjects with MetS have a decreased HRV. None of the previous studies have assessed the complex relationship between HRV and PhA. To the best of our knowledge, we are first to analyze this relationship.

Besides the influence of PhA on HRV, there are other time-related HRV fluctuations associated with autonomous cardiac control, which depends on many factors including circadian activity, resting, and sleeping-waking cycles. To eliminate possible physical activity-related fluctuations, HRV variability often is limited to short-term office assessment. However, this obscures the real picture for the obvious reason that such an assessment does not take into account other daily fluctuations indicated above. While a more comprehensive HRV variability testing in an outpatient setting has been previously reported [27], there are many unresolved methodological issues, especially with respect to the elimination of the influence of SNS. During 24 h of ambulatory monitoring, subjects behave freely and are exposed to various stressors [28,29] which elevate HR up to 110-120 bpm. HRV is influenced by physical activity which increases SNS activity, leading to the elevation of HR and a reduction of HR fluctuations. In the outpatient setting, a subject is exposed to a number of confounding factors, and the averaging of HRV indicators conceals them. Filtering RRI out or adjusting for confounding factors has been an issue for different reasons previously ignored in a number of published studies. 

Using 24 h ambulatory monitoring data, we demonstrated that SNS activity increase reduces HRV both in the control and even more in the MetS group subjects. Manfredi et al. [30] showed that HRV is inextricably linked to HR in many different situations: the lower the HR, the higher the HRV. In addition, many authors raise the question of whether HRV metrics should be “corrected” for the mean heart period or its inverse metric, heart rate [31,32,33]. Our findings show that either can be used and suggests some needed adjustments for 24 h monitoring for increasing the validity of the test.

Physical exercise, by its nature, always causes a long-lasting HR reaction, as sympathetic heart control acts as a low-frequency filter capable of replacing HR modulation with a lower than 0.15 Hz frequency. Unlike short-term HRV analysis, a long-term HRV analysis, especially in the VLF and LF band, is challenging because of the difficulty to synchronize PhA and RRI. Failure in well-calibrated synchronization does not allow the test to be specific and sensitive enough. On top of synchronization failure, possible past drawbacks include the scope of the physical activity interference with the end results, backed up by the fact that physical activity almost always activates SNS and increases HR [29] accordingly.

Aoyagi et al. [19] showed that body movements affect HRV. They cause both long-lasting and short-term phase changes in HR. From these, as well as from other published results, it can be inferred that all spontaneous movements should be controlled in upcoming studies [34]. However, the question remained which movements need to be taken into account, and how to control for them. Besides the aforementioned synchronization challenge, our study has addressed this issue as well.

In order to more accurately evaluate HRV in our study, ARX based modeling was performed and the effect of day-time physical exercise on HRV has been eliminated. The obtained results confirm that accurate synchronization of actigraph data with each RRI allows us to more accurately assess the effect of physical load on HRV variables. In addition, physical activity was found to be strongly related to the lower spectrum bandwidth and time-domain indicators of HRV. In concord with the data of Zulfiqar et al. [35], we have demonstrated that the use of a Holter ambulatory monitoring alone is not sufficient for the comprehensive evaluation of individual cardiovascular reactions.

Yuda et al. [12] used a simple regression model to measure HRV (SDNN) and accelerometer readings in four periods of the day, and only day-time physical activity was found to have a positive effect on SDNN, resulting in only a 7% SDNN difference between individuals. In agreement with the published data, we once again demonstrate that night-time HRV assessment is not altered by the physical activity: therefore, further adjustments are of little value. Though the challenges of adjusting HRV for confounding factors could be largely avoided by performing the test in an inpatient setting or limiting it to the night-time, such an approach would have a major limitation of being detached from the daily variations of the HRV present in real life. By performing a more comprehensive 24 h assessment, which takes into account the role of ANS on HRV, our study has taken a major step towards transcending this limitation.

## 5. Conclusions 

Simultaneous monitoring of physical activity and RRI along with the polynomial autoregressive modeling enables us to exclude the influence of physical activity on day-time HRV. Prior to the elimination of the influence of physical activity, individuals with MetS (50–54 years old) were determined to have blunted HRV. After the elimination of the influence of physical activity, MetS was found to be associated with the less pronounced short-term HRV indices (RMSSD, pNN50, SDNN index, SD1) and low-frequency spectral components. Physical activity elimination made it possible to differentiate the sympathetic nervous system confounders and revealed that MetS is associated with a mitigated parasympathetic tone. Consequently, we suggest that actigraphy ought to be used to monitor and exclude HRV variance associated with physical activity, thus reducing the impact of this potentially confounding factor.

## Figures and Tables

**Table 1:** Demographic, hemodynamic and clinical data.

Variable	Control Group (37)		MetS Group (69)	
Male	16 (43.2%)		38 (55.1%)	
Female	21 (56.8%)		31 (44.9%)	
Age (years)	51.7 ± 1.38		52.9 ± 1.14 **	
Weight (kg)	74.9 ± 11.3		89.5 ± 13.9 **	
WC (cm)	86.1 ± 8.87		102.5 ± 10.08 **	
Abdominal obesity (%)	5.4		87.0 **	
Overweight (%)	10.8		97.1 **	
BMI (kg/m^2^) (%):				
Normal (18.5–24.9)	55.6		2.9 **	
Overweight (25.0–29.9)	41.7		43.5 *	
Obesity (30.0–40.0)	2.8		53.6 **	
SBP (24 h)	118.5 ± 8.8		127.9 ± 10.7 **	
DBP (24 h)	73.7 ± 6.7		78.9 ± 8.1 **	
SBP (diurnal/nocturnal)	124.1 ± 8.9	106.5 ± 10.1	134.1 ± 11.2 **	114.8 ± 11.6 **
DBP (diurnal/nocturnal)	77.9 ± 8.2	63.8 ± 7.2	83.8 ± 8.9 **	69.1 ± 8.7 **
Glucose concentration (mmol/L) (increase, %)	4.82 ± 0.57 (0.0%)		5.72 ± 0.80 (55.1%) **	
Dyslipidemia:				
TC (mmol/L)	5.61 ± 1.2		6.39 ± 1.3 *	
LDL-C (mmol/L)	3.62 ± 0.99		4.2 ± 1.2 *	
TG (mmol/L)	1.0 ± 0.39		2.17 ± 1.1 **	
HDL-C (mmol/L)	1.69 ± 0.46		1.4 ± 0.5 *	
Dyslipidemia (%)	64.9		98.6 **	
Diabetes (%)	0.0		14.5	
Metabolic Syndrome (%)	0.0		100	

* *p*-Value < 0.01 and ** *p* < 0.001 between control and MetS groups; data are shown as means ± standard deviation. BMI: Body Mass Index; WC: Waist Circumference; BP: Blood Pressure; SBP: Systolic Blood Pressure; DBP: Diastolic Blood Pressure; TC: Total Cholesterol; LDL-C: Low-Density Lipoprotein Cholesterol; HDL-C: High-Density Lipoprotein Cholesterol; TG: Triglycerides.

**Table 2:** Heart rate variability data.

HRV Variables	Diurnal		Nocturnal	
	Control	MetS	Control	MetS
***RRI (ms)***	734.0 ± 82.7	762.3 ± 99.6	951.1 ± 100.0 ###	953.0 ± 112.4 ###
*Time-domain variables*				
*SDNN (ms)*	107.7 ± 26.6	105.5 ± 27.7	93.0 ± 31.1 #	90.0 ± 26.4 #
*SDNN index (ms)*	53.8 ± 14.1	47.8 ± 13.0 *	56.7 ± 16.0	54.3 ± 16.7 #
*SDANN (ms)*	91.3 ± 26.9	92.5 ± 26.8	63.2 ± 20.0 ###	64.4 ± 20.9 ###
*SD1 (ms)*	14.2 ± 5.4	12.9 ± 4.9	19.9 ± 8.4 ###	19.7 ± 8.3 ###
*SD2 (ms)*	151.6 ± 37.5	147.9 ± 37.0	128.8 ± 40.7 #	125.1 ± 35.9 ##
*RMSSD (ms)*	20.0 ± 7.5	18.3 ± 6.9	27.9 ± 10.7 ###	27.8 ± 11.8 ###
*pNN50 (%)*	3.8 ± 3.5	3.2 ± 3.8	8.9 ± 8.6 ###	9.4 ± 9.5 ###
*Frequency-domain variables*				
*VLF (ln ms^2^)*	7.47 ± 0.55	7.27 ± 0.53	7.69 ± 0.59	7.52 ± 0.58 #
*LF (ln ms^2^)*	5.86 ± 0.61	5.51 ± 0.59 **	5.89 ± 0.58	5.63 ± 0.63 *
*HF (ln ms^2^)*	4.66 ± 0.77	4.50 ± 0.81	5.24 ± 0.87 ##	5.13 ± 0.92 ##
*LF/HF*	3.62 ± 1.35	3.04 ± 1.37 *	2.15 ± 1.03 ###	1.95 ± 1.16 ###

* *p* < 0.05; ** *p* < 0.01; *** *p* < 0.001, between control and MetS group; # *p* < 0.05; ## *p* < 0.01; ### *p* < 0.001 between diurnal and nocturnal respectively. Data are presented as means ± standard deviation. RRI: R-R intervals; SDNN: standard deviation of all normal to normal RR intervals; SDANN: standard deviation of 5-min average RRI; RMSSD: the square root of the mean of the sum of the squares of differences between adjacent RRI; SDNN index: mean of the standard deviation of all 5-min RRI segments in 24 h; SD1: short-term beat-to-beat RRI variability from the Poincaré plot (width); SD2: long-term beat-to-beat RRI variability from the Poincaré plot (length); pNN50: the percentage of intervals >50 ms different from preceding interval; VLF: very low frequency band (0.003–0.04 Hz); LF: low frequency band (0.05–0.15 Hz); HF: high frequency band (0.15–0.5 Hz); LF/HF: ratio between LF and HF power.

**Table 3:** Heart rate variability data after elimination of physical activity influence.

Variables	Control (37)	MetS (69)	Control (37)	Mets (69)
	Mean ± SD	Mean ± SD	Delta Mean	Delta Mean
*RRI (ms)*	785.96 ± 93.03	819.61 ± 104.42	−62.3 ###	−75.6 ###
*Time-domain variables*				
*SDNN (ms)*	91.30 ± 27.67	96.38 ± 33.85	6.2	7.2
*SDNN index (ms)*	59.86 ± 14.89	50.31 ± 16.21 *	−8.39 #	−9.24 #
*SDANN (ms)*	65.44 ± 29.53	77.67 ± 36.93	13.20	13.30
*SD1 (ms)*	18.47 ± 5.32	15.34 ± 5.24 **	−4.32 ###	−3.02 ###
*SD2 (ms)*	127.65 ± 39.25	135.37 ± 47.78	9.46	10.54
*RMSSD (ms)*	26.11 ± 7.52	21.70 ± 7.41 **	−6.11 ###	−4.26 ###
*pNN50 (%)*	7.81 ± 5.93	4.72 ± 4.92 **	−3.87 ###	−1.82 ###
*Frequency-domain variables*				
*VLF (ln ms^2^)*	7.43 ± 0.59	7.12 ± 1.25	−0.104	−0.102
*LF (ln ms^2^)*	5.65 ± 0.63	5.07 ± 1.46 **	0.180	0.245
*HF (ln ms^2^)*	3.18 ± 0.81	2.83 ± 1.61	1.483	1.494

* *p* < 0.05; **p < 0.01; *** *p* < 0.001, between control and MetS group. # *p* < 0.05; ### *p* < 0.001, before and after elimination of PhA. Data are presented as means ± standard deviation. RRI: R-R intervals; SDNN: standard deviation of all normal to normal RR intervals; SDANN: standard deviation of 5-min average RRI; RMSSD: the square root of the mean of the sum of the squares of differences between adjacent RRI; SDNN index: mean of the standard deviation of all 5-min RRI segments in 24 h; SD1: short-term beat-to-beat RRI variability from the Poincaré plot (width); SD2: long-term beat-to-beat RRI variability from the Poincaré plot (length); pNN50: the percentage of intervals >50 ms different from preceding interval; VLF: very low frequency band (0.003–0.04 Hz); LF: low frequency band (0.05–0.15 Hz); HF: high frequency band (0.15–0.5Hz); LF/HF: ratio between LF and HF power.
